# Social network-based ethical analysis of COVID-19 vaccine supply policy in three Central Asian countries

**DOI:** 10.1186/s12910-022-00764-1

**Published:** 2022-03-09

**Authors:** Timur Aripov, Daniel Wikler, Damin Asadov, Zhangir Tulekov, Totugul Murzabekova, Kerim M. Munir

**Affiliations:** 1 Department of Public Health and Healthcare Management, Tashkent Institute of Postgraduate Medical Education, Parkent str. 51, Tashkent, 100007 Uzbekistan; 2 Department of Global Health and Population, Harvard School of Public Health, 677 Huntington Avenue, Boston, MA 02115 USA; 3 Al-Farabi Kazakh National University, 71 Al-Farabi Avenue, Almaty, 050040 Kazakhstan; 4 Kyrgyz-Netherlands Community of Volunteers Tuberculosis Foundation, Zhantosheva 121/33, Bishkek, Kyrgyzstan; 5 Harvard Medical School, Boston Children’s Hospital, Developmental Medicine Center, ​300 Longwood Ave, Boston, MA 02115 USA

**Keywords:** COVID-19, Vaccine, Bioethics, Low- and middle-income countries, Central Asia

## Abstract

**Background:**

In the pandemic time, many low- and middle-income countries are experiencing restricted access to COVID-19 vaccines. Access to imported vaccines or ways to produce them locally became the principal source of hope for these countries. But developing a strategy for success in obtaining and allocating vaccines was not easy task. The governments in those countries have faced the difficult decision whether to accept or reject offers of vaccine diplomacy, weighing the price and availability of COVID-19 vaccines against the concerns over their efficacy and safety. We aimed to analyze public opinion regarding the governmental strategies to obtain COVID-19 vaccines in three Central Asian countries, focusing particularly on possible ethical issues.

**Methods:**

We searched for opinions expressed either in Russian or in the respective national languages. We provided data on the debate within three countries, drawn from social media postings and other sources. The opinion data was not restricted by source and time. This allowed collecting a wide range of possible opinions that could be expressed regarding COVID-19 vaccine supply and human participation in the vaccine trial. We recognized ethical issues and possible questions concerning different ethical frameworks. We also considered scientific data and other information, in the process of reasoning.

**Results:**

As a result, public views on their respective government policies on COVID-19 vaccine supply ranged from strongly negative to slightly positive. We extracted the most important issues from public debates, for our analysis. The first issue involved trade-offs between quantity, speed, price, freedom, efficacy, and safety in the vaccines. The second set of issues arose in connection with the request to site a randomized trial in one of the countries (Uzbekistan). After considering additional evidence, we weighed individual and public risks against the benefits to make specific judgements concerning every issue.

**Conclusions:**

We believe that our analysis would be a helpful example of solving ethical issues that can arise concerning COVID-19 vaccine supply around the world. The public view can be highly critical, helping to spot such issues. An ignoring this view can lead to major problems, which in turn, can become a serious obstacle for the vaccine coverage and epidemics’ control in the countries and regions.

## Background

For many low- and middle-income countries (LMICs), equitable access to COVID-19 vaccines during the current pandemic becomes essential for avoiding catastrophic loss of life, health, and for their economic wellbeing. According to the Eurasia Group report, the COVID-19 vaccines will generate economic benefits up to 466 billion US dollars by 2025 among the ten major economies [1]. The pandemic has caused huge devastation even in some of the wealthy countries, despite their high ranks for epidemic preparedness. For the LMICs, the emergency presents an even greater burden, as they do not have resilient public health systems, including limited capacity for intensive care, and they cannot afford to maintain living standards through government payments.

With the fast-moving pandemic, the COVID-19 Vaccine Global Access (COVAX) initiative has raised hopes for immunization against the coronavirus in LMICs. However, high-income countries have secured future supplies of vaccines, and the access for the rest of the world became uncertain [2]. As WHO reported, richer countries had received more than 87%, and low-income countries just 0.2% from all vaccine doses that have been administered globally [3]. Any priority-setting rule for allocating vaccines among LMICs would invite ethical debate, and this has been the case for COVAX’s, which tracks population size over other indicators of acute need [4, 5]. But the importance of this ethical choice for LMICs is overshadowed by the serious shortfall of vaccines available to COVAX for distribution.

With such restrictions, each of LMICs must develop a strategy for obtaining and allocation of COVID-19 vaccines to avoid disaster. Understandably, the governments of LMICs and world pharmaceutical companies were interested in direct contacts to supply their communities with the necessary doses of the vaccine. This could guarantee faster profit for vaccine producers and less vaccination cost for recipient countries, leading to higher overall public health benefits [6]. Several LMICs have received their first vaccine shipments not from COVAX and world pharmaceutical companies, but from China and Russia, countries that use the vaccines to expand their influence [7]. Although China withheld claiming high efficacy of its Sinovac or Sinopharm vaccines until the end of phase III trials, Russia declared its Sputnik V vaccine at 95% efficacy, basing on preliminary results [8]. Both countries have actively offered early and low-cost supply of their vaccines round the world, sometimes proposing the recipient countries to provide a human subject site for randomized controlled vaccine trials. The reason, in addition to economic and political ones, has been the need to get data about long-term safety of their COVID-19 vaccines. Although these sites have included highly populated countries such as Brazil, they have also included neighboring countries, such as Uzbekistan. The governments in these countries have faced difficult decision whether to accept or reject offers of vaccine diplomacy, weighing price and availability of COVID-19 vaccines against concerns over their efficacy and safety.

The primary objective of our study was to analyze public opinion regarding the governmental strategies to obtain COVID-19 vaccines, in three post-Soviet Central Asian countries: Uzbekistan, Kyrgyz Republic (Kyrgyzstan) and Kazakhstan. Public participation in the policy choice will provide an important voice of those who are directly affected by the outcomes, contributing to the ethical soundness of the resulting choices [9].

## Methods

Data was collected on a range of opinions expressed by members of the public as well as by politicians, officials, and experts. We considered reports and messages from web pages of leading newspapers, TV channels and social media outlets. The opinion data was collected regarding a specific period but was not restricted by source. This allowed collecting a wide range of possible opinions that could be expressed, regarding COVID-19 vaccine supply and the public’s participation in vaccine trials. The initial search for these data from three countries relied on the Google search engine. This was supplemented with data from Facebook pages of news media, which are popular in three countries, such as Radio Liberty, Gazeta.uz, Sputnik and others.

The Russian equivalents of the following English keywords and their combinations were used for the search: “COVID-19, vaccine, Uzbekistan”; “COVID-19, vaccine, Kazakhstan” and “COVID-19, vaccine, Kyrgyzstan”. We searched opinions expressed either in Russian or in the respective national languages (Uzbek, Kazakh, and Kyrgyz). We used a meaning-based (abstractive) approach to summarize public opinions on vaccine supply and participation in vaccine trials. This helped to consider every view from different sources with different language use [12].

### Inclusion criteria

We decided to include postings from people living in Uzbekistan, Kyrgyz Republic, and Kazakhstan at the time of posting. This could contribute more to engaging the voices of those who are directly affected by the policy of vaccine supply in their countries. We included postings done in the period from May to December of 2020. Because of global travel restrictions, few of these postings could originate from outside the boundaries of these countries. The included postings were not restricted by ethnicity, residency, age, sex, and social status of their authors.

### Ethical analysis

We sought debates over COVID-19 vaccine supply and human participation in the vaccine trials in the Central Asian countries. We engaged debates that offered ethical views and reasoning, as opposed to disputes over theology, ideology, or law. For the analysis, we recognized ethical issues with possible questions to solve them concerning three different value-based ethical frameworks [13, 14]. These frameworks combine both normative and applied approaches and provide high applicability to solve specific issues. We considered scientific data and other available information, in the process of reasoning. As internationally-accepted ethical standards, we applied the UN’s Declaration of Human Rights (UDHR) principles [15] and, for the case of health-related research involving human subjects, guidelines prepared by the Council for International Organization of Medical Sciences (CIOMS) [16]. For every ethical issue with questions, we developed the inference as the final judgement.

## Results

We considered all publicly available reports and messages from media web pages. They included statements, views, and arguments directly or indirectly related to COVID-19 vaccine supply, the policy of vaccination, and human participation in the vaccine trial. A categorical description of the views concerning discussed issues, source of views, and countries is provided in Tables 1 and 2.

**Table 1:** Number and distribution of views concerning discussed issues

	Uzbekistan	Kazakhstan	Kyrgyz Republic	Total
Vaccine import	21	36	18	75
Manufacturing local vaccines	7	11	9	27
Mandatory versus voluntary vaccination	13	22	19	54
Participation in the vaccine trial	22	9	7	38
Others	3	5	6	14

**Table 2:** Number and distribution of views concerning their sources

	Uzbekistan	Kazakhstan	Kyrgyz Republic	Total
Government officials and politicians	6	11	3	20
Experts	4	6	5	15
Public (non-experts) Male Female Undefined	68 36 21 11	54 28 23 3	51 23 20 8	173 87 64 22

Most views in social media were expressed by the public with just some examples of views provided by government officials and experts. The most frequently discussed issue was the quality of the vaccines offered by China or Russia with capacities for their local production. Governmental sources declared a high probability of using vaccines from these countries, for the mass vaccination. In Kazakhstan, local experts are concerned about the problem of the big size of their country to maintain a cold chain for storage of the vaccine, with a low possibility to use vaccines other than Russia’s. The public view also concentrated on the choice between countries rather than manufacturers, with some touch of available evidence about vaccines’ efficacy and safety. Another discussed issue was mandatory vs. voluntary strategies with stressing possibilities of human rights violations at the first one.

“For whom you do this, for the people or for your own or political interest…the people should have a choice: who wants Chinese please, who wants Russian please… (middle-age man, Uzbekistan)

Putin himself advertised this after vaccination re-infection is possible, but the disease proceeds in a mild form without complications and without health consequences (undefined user, Kyrgyzstan)

The problem is precisely the quality of the vaccine (middle-age man, Kazakhstan)

No one can tell me what medicine to inject me (young man, Uzbekistan)

Another critical issue became the policy of recruiting human subjects for COVID-19 vaccine trials. Specifically, the public view concerned the decision of the government in Uzbekistan to provide a site for a phase III trial of the Anhui Zhifei Longcom Biopharmaceutical’s (China) vaccine. The Uzbek officials declared that participation of 5,000 subjects in the trial would be voluntary and base on participants’ formal consent. The authorized ministry of Uzbekistan stressed that its workers and their families are also taking part in this trial as subjects. However, most of those who commented on the trial in the media claimed that Chinese vaccine producers may want Uzbek people to be “guinea pigs” for their trials. They hold that Uzbek people should get only vaccines with proved effectiveness and safety. Only some postings accepted that there is nothing bad in taking part in such trial, especially if it results in an early vaccine supply.

…and the Chinese one has not been tested on anyone at all, and our people are worse than monkeys to test on it? And at the expense of yourself prick yourself, so you bent it! I don't need any! All is made (Russian slang) in an emergency mode, it is still unknown how later this vaccine will come back! (young woman, Ferghana, Uzbekistan)

Are Uzbek people rabbits to be tested by Chinese and Russians? Let them stupid, who signed such agreement, to take part on such testing. Do not touch ordinary people (middle-age woman, Uzbekistan)

Let the Uzbek vaccine be used exclusively for officials … (young man, Uzbekistan)

Postings by politicians and experts across the three countries predominantly supported the views of their governments. Public views remained evenly divided regarding their respective government policies and ranged from strongly negative to neutral or slightly positive.

## Ethical issues

The principal ethical issues stressed on discussions within three countries, over strategies to obtain COVID-19 vaccines, arose in two categories. Ethical concerns included the acceptability of putting their citizens at possible risk so that the population can gain access to the vaccines it needs; and ethical issues in the conduct of the trial.

### Ethical issue #1

The Central Asian countries seek inexpensive and easily-administered strategies to provide safe and effective COVID-19 vaccines for their communities. The COVAX program is designed to provide vaccines that proved their quality in phase III trials and/or have been approved by WHO for emergency use. However, this source of vaccines can meet at most 20% of every country’s needs. For the remainder, three countries are on their own; and this is when the ethical choices arise. Their governments declared plans to import vaccines from Russia or China and even produce them internally. However, the efficacy and safety of COVID-19 vaccines from specific manufacturers may not be enough, so can even harm countries’ communities. With the aim to provide faster benefit for more people, governments can choose mandatory over voluntary vaccination policy, and this will become another ethical choice.

## Questions:

1. What kind of vaccine coverage and allocation strategy could be justifiable to provide access to vaccine in highly restricted supply conditions?
2. Is this justifiable to import COVID-19 vaccines only from Russia or China and produce them locally despite high concerns of these vaccines’ safety and efficacy?
3. Is a mandatory vaccination campaign justifiable, considering the possibility of low efficacy in used vaccines and significant human rights violations?

### Ethical issue #2

A provision of human subjects for the Zhifei Longcom’s vaccine phase III trial led to a privileged access to this COVID-19 vaccine for Uzbekistan. This guaranteed a faster supply of the vaccine for more people in the country. However, the vaccine being tested can potentially harm subjects in a short or long term, even leading to their death. In this way, its clinical efficacy possibly will not outweigh its potential risk. A level of ethical expertise, in the country, raises worries about the high risk of misconduct during the trial and violation of the rights of its subjects.

## Questions:

1. Is it acceptable to provide subjects for testing Chinese Zhifei Longcom’s vaccine to guarantee faster benefit for the subjects of the trial and all community in Uzbekistan?
2. To what extent ethical requirements to protect human subjects are being followed in the trial conducted in Uzbekistan?

## Discussion

Development of local strategy in obtaining and allocating COVID-19 vaccines as well as in testing them has become a not easy task. This is, to our knowledge, the first study that analyzed public opinion regarding such strategies in low- and middle-income countries. We offered evidence of the debate within three Central Asian countries, drawn from web pages and social media postings. This debate arose ethical issues and questions over which there was considerable disagreement within each country. We will provide possible solutions for these choices by making practical inferences on them.

### Ethical issue #1

There is lack of reliable data from Central Asian countries concerning the herd immunity to SARS-CoV-2 in their communities. Data on other coronaviruses suggest that individual protection might be short lived; however, goes to 12–18 months in duration [17]. Whether past infection prevents severe COVID-19 is still not clear. However, vaccines can sharpen immunity in previously infected people and contribute to mild illnesses in non-infected ones [18]. A spread of mutant strains that are more contagious and fatal [19] may contribute to reducing lockdowns effects and to the rise a role of the vaccination in the epidemic control. By this time, the Delta variant looks deadliest [20], and it reached Central Asia. The Omicron strain is suggested to be milder [21]; however, it is not clear how it will impact on the region. This proves the need for the mass campaign as a way to provide individual protection, in all three countries. Two of them, Uzbekistan and Kyrgyzstan, started their campaigns in April 2021, and Kazakhstan declared its start even earlier. Up to 2022, there are 137 candidate vaccines in clinical and 194 in preclinical development, and 24 of them are within the WHO evaluation process [22, 23]. And an assessment to use for vaccination was finalized for ten vaccines, with no Russian vaccines on that list. However, the formal report about the phase III trial of Sputnik V declared its efficacy at 91% [24]. Until now, Chinese Sinopharm and Sinovac got WHO approval for emergency use with the respective 79% and 51% efficacy for preventing symptomatic cases [25, 26].

For countries with a larger population, such as Uzbekistan, low efficacy can make a higher negative impact. An estimated threshold for COVID-19 vaccine efficacy, in case of full vaccination coverage, is about 60% at reproduction number (*R*_0_) equal to 2.5–3.5 [27]. The efficacy threshold rises to 70% when coverage drops to ^3^/_4_ and rises to 80% when coverage drops to ^3^/_5_ of the community. This makes countries more interested in higher vaccination coverage in a case of lower vaccine efficacy. The most difficult is how to define target coverage in the case of a variety of vaccines with different efficacy. By this time, one of the lowest declared efficacy rate (70%), among known candidates, is in the Oxford-AstraZeneca vaccine [28]. This means that countries will need ^3^/_4_ vaccination coverage in case they use only such vaccine as the sole intervention. If consider that other applied vaccines may have higher efficacy, this coverage rate would be enough for a maximum public benefit. The cost of Sputnik V per patient is about half of the cost of the Pfizer/BioNTech vaccine [29]. Chinese vaccines are comparable in cost with those from Western producers. A good point of non-Western vaccines is that they don’t require subzero storage, so they are better suited for mass use in warmer conditions. However, the Oxford-AstraZeneca vaccine can also be stored at regular fridge temperature.

The selection of COVID-19 vaccines for the mass campaign, in Central Asian countries, as well as their choice for every individual use raises serious ethical issues. Around the world, government officials advocate and demonstrate their adherence to COVID-19 vaccination. This also looks like the way to demonstrate that they would share individual risk and benefit with their communities, in times of unclear or insufficient evidence about vaccines. However, most of such examples come from developed countries in which officials and communities are supposed to be vaccinated with the same vaccines, leading the similar risk and benefit among them. In Central Asia, vaccines’ deficit and potentially higher range in their efficacy and safety can lead to unequal distribution of individual risk and benefit, with the possibility of an abuse of power. Low transparency can contribute to unjust access to vaccines, leading to the violation Article 21(2) of UDHR. Our data proves a low public trust in governments, and social media users claimed their governments to publicly demonstrate their preparedness to get vaccinated with the same COVID-19 vaccines as used for the mass campaign, that is to share the risk. The public trust can fall even more if the numbers of side effects after use of the specific COVID-19 vaccines grow or in case of their low effectiveness. An important factor becomes whether equal access to healthcare will be guaranteed for all, in case of side effects or unexpected conditions. Communities cannot expect such justice if their previous access to healthcare was highly dependent on their social status. In case the public does not see the response to these issues, the current situation can lead a vaccine rejection or hesitancy in Central Asia, where communities historically were highly adherent to vaccination. The application of prioritization strategies that propose to vaccinate specific sub-communities such as the elderly, live-saving or social service staff, and close contact individuals [30, 31] will probably fail in this course.

From this view, the employment of mandatory vaccination looks inappropriate, in Central Asia. We considered four additional conditions to make this decision [32]. First, a lower proportion of the older population and lower than in Europe and US fatality rates make threat for public health less grave, in three countries. Kyrgyzstan has the highest case fatality (1.71%) in a smaller population, and this can be due to the policy of registration of all unclear deaths as COVID-19, at the peak of the epidemic. However, estimates of the Institute for Health Metrics and Evaluation consider Central Asia as a region with the highest ratio of total COVID-19 deaths to reported deaths [33]. An issue can be that all three countries had relatively low coverage by PCR testing, due to its high cost [34]. This can be a factor both increasing and decreasing the true epidemics burden for the countries. Second, even evidence from phase III trials for some vaccines, there are common both experts and public concerns that vaccine testing has been rushed, and they may not be as safe and effective as they are declared. And this issue relates more to Russian and Chinese vaccines. This condition can decrease public and individual benefits and increase individual risks from the vaccination, in three countries. Third, a systematic review of observational studies confirmed the effectiveness of social distancing and wearing masks in reducing COVID-19 transmission [35]. This means that they still have high potential as alternatives to vaccination, especially in communities where they were not widely applied. Finally, another problematic issue is how proportionate coercion will be in a case of mandatory vaccination policy. In addition to unequal individual risk distribution, one can expect that coercion will be too strict, leading to significant human rights violations and social restrictions.

### Inference

The policy of COVID-19 vaccines supply, oriented to their imports only from specific countries (Russia or China) or producers cannot be justifiable this time. The market is developing fast and new vaccines appear to be available for mass campaigns in Central Asian countries. Their governments should consider their efficacy rates as a primary factor for choice in planning the vaccination coverage. This means that countries are recommended to use vaccines with at least 70% efficacy; in this case, they would need to provide ^3^/_4_ coverage by vaccination. The countries can use vaccines at a lower cost, in case they have this or higher efficacy rates. The cost will also include expenses for storage and transportation of vaccines. The government policy should be transparent enough to inform communities about the way how equal sharing of individual risks and benefits will be provided. This way can contribute the increase of public trust in governments and raise vaccination coverage. To prevent inequality in access to vaccines having different efficacy and safety rates, we would recommend, for every individual use to make a random selection from the list of vaccines available in the country.

### Ethical issue #2

As an emergency way to test their vaccines, all manufacturers conduct clinical trials around the world, especially considering countries with big populations and high COVID-19 morbidity. Only Uzbekistan, in Central Asia, provides subjects for phase III trial of the Chinese Zhifei Longcom’s vaccine. And this time, it becomes the main vaccine used for a mass campaign in Uzbekistan. However, under the WHO evaluation process, it is still marked as in the step of expression of interest. The Uzbek study is a component of the trial that is registered in ClinicalTrials.gov as an international multicenter study (ID: NCT04646590) with total 18 study locations and requiring informed consent from all human subjects. This time it is clear that a previously planned Uzbek sample size (5000 subjects) doubled, presumably, due to the fall in COVID-19 morbidity in the country or other locations. This means the number of subjects at risk has also doubled, while individual benefit from this vaccine testing was very low for these subjects. The reason is that COVID-19 is most fatal for older people having chronic conditions and their risk factors [36]. However, the trial involved only healthy young and middle-aged subjects genuinely having a low risk of severe COVID-19 in case it would develop on them. An authorized ministry, in Uzbekistan, declared that the vaccine was safe because it is recombinant and does not contain the virus. It is true that this type of vaccines has reduced side effects [37]; however, its safety concerns not only viral target but also a variety of other ingredients and by-products of manufacturing. An unclear safety and efficacy make the public benefit from use of this vaccine highly questionable as well.

From this view, it looks unreasonable to provide a site for the trial as a way to get better access to the vaccine for the subjects of the trial. Moreover, the country should not guarantee the participation of a specific number of subjects on the trial. The country’s role should be limited to formal permission to recruit subjects. The most important for the country is protecting the rights of the subjects, irrespective of what public benefit is expected to get from the tested vaccine. There is no reliable data about the text of informed consent and to what degree the process of its collection complies with Guidelines 9 and 10 of CIOMS. From some sources it become clear that there were many government military workers among participants, so they could be vulnerable from the view of considering their rights as subjects of the trial. According to officials, the Ethics Committee at the Ministry of Health approved conducting this trial in the country. They also declared that the study would be stopped in case adverse effects will go in 30% of the human subjects. However, a big issue becomes how transparent was the committee’s work and to what degree it was independent in its decisions.

### Inference

A potentially low individual benefit could not outweigh the risk for subjects involved in Zhifei Longcom’s vaccine phase III trial, in Uzbekistan. The policy to provide a site for testing any specific COVID-19 vaccine could be justified only in a case the trial guaranteed the protection rights of every subject. The Ethics Committee at the Ministry of Health should have provided careful monitoring of the study, and it should have had a right to stop the trial in any step. The informed consent should have been delivered properly to subjects, leading to their free agreement to participate in the study. By this time, the clinical efficacy and safety of Zhifei Longcom’s vaccine are unclear even relating to Uzbek participants of the trial. These findings become a critical point for a decision to keep using this vaccine in Uzbekistan and for plans to provide coverage level.

## Limitations

This ethical analysis is not based on the exact representation of public opinions related to COVID-19 vaccine supply or participation in the vaccine trial, in Central Asia. The proportion of communities that do not use social media or have steady Internet access can be high and can vary among countries. There were no ways how to collect data in such communities during the epidemic. The idea was to collect all possible ranges of attitudes, from three countries, to adjust them with the basic ethical principles in the process of reasoning. The quality of social media data was not previewed and this could decrease the quality of the analysis. Specific issues and questions extracted and discussed in our analysis can be distinct from the other world, and this can limit the generalizability of our analysis. However, issues concerning COVID-19 vaccine supply become principal for every country, this time. The epidemic’s patterns and curves can change unpredictably in three countries, leading to higher COVID-19 fatality rates. This would increase the public risk causing higher benefits from mandatory vaccination. However, this way will not resolve problems arising from the unequal risk and benefit distribution during the mass campaign.

## Conclusions

Despite the limitations of our study, we believe that it would be a helpful example of solving ethical issues that can arise concerning COVID-19 vaccine supply around the world. All countries should weigh risks and benefit from specific policies and stay very sensitive to the possible harm. The public view, even not based on strong knowledge and expertise, can be highly critical, helping to spot such issues. An ignoring this view can lead to major problems, which in turn, can become a serious obstacle for vaccine coverage and epidemics’ control in the countries and regions.

## Data Availability

The data analyzed are available in Internet and Facebook pages of news media, such as Radio Liberty, Gazeta.uz, Sputnik and others.

## Abbreviations

CIOMS: Council for International Organization of Medical Sciences
COVID-19: CoronaVirus Disease 2019
COVAX: COVID-19 Vaccine Global Access
LMICs: Low- and Middle-Income Countries
SARS-CoV-2: Severe Acute Respiratory Syndrome Coronavirus 2
PCR: Polymerase Chain Reaction
UDHR: United Nations Declaration of Human Rights
WHO: World Health Organization
